# On Evidence in Support of Male Circumcision in HIV Prevention: What Next?

**DOI:** 10.1371/journal.pmed.0030066

**Published:** 2006-01-31

**Authors:** Adamson Sinjani Muula

**Affiliations:** **1**University of North Carolina Chapel Hill, Chapel Hill, North Carolina, United States of America and University of Malawi College of Medicine, Zomba, Malawi

The study by Auvert et al. [ 1] will certainly go into the history of HIV prevention as a landmark. The study is important because the results are the first blinded randomized study demonstrating that HIV can be prevented by male circumcision (MC). Double-blinded studies are considered to be the gold standard in research but because of the nature of this intervention, double blinding was impossible—i.e., it was impossible for the men to be circumcised without them knowing that they had been circumcised.

The study suggests that MC could join the interventions for HIV/AIDS already available—i.e., highly active antiretroviral therapy (HAART), short-course antiretroviral therapies and caesarean section in preventing mother-to-child transmission, postexposure prophylaxis, condoms, abstinence, and treatment of sexually transmitted infections (STIs). Like many other health interventions, MC (if its effectiveness is further demonstrated in subsequent randomized studies and then adopted within national policies) will be indicated and suitable for some people, but not for others, for a variety of reasons. It would therefore be unfortunate if we were to start promoting MC at the expense of other intervention measures. The authors did not suggest that we should do so, but there is always the danger that some people will seek to boost efforts on one intervention whilst neglecting others.

The other challenge is that medical practice is conservative—i.e., it is unlikely that any country will immediately include MC in its policy for the prevention of HIV. The reasons include the following: these findings may not be corroborated in forthcoming studies and the potential harms still need to be considered in order to assess the cost–benefit ratio. Even in South Africa where the study was carried out, it will be a while before MC is incorporated into the national HIV prevention policy. Interestingly, however, the institutional review board stopped the study prematurely—as is always deemed ethical—suggesting implicitly perhaps their endorsement of MC and that it ought to be standard practice.

The authors indicate that “to wish to be circumcised” was one of the inclusion criteria. It is not clear to me what “wishing” meant—i.e., was it that they found MC acceptable or that they wanted to be circumcised but, for some reason, had not had the opportunity? If the interpretation is the latter, it would be important later to identify the barriers to MC that may operate in countries in southern Africa. Knowledge of these barriers will inform the policy debates.

While the policy debates rage, the scientific community has an enormous responsibility—i.e., ensuring that well-conducted studies are carried out in other settings to either confirm or dispute the findings. Results from other settings will be awaited with eagerness.

Researchers in the HIV field face the dilemma of not subjecting their study subjects to undue harm through stigmatization and discrimination. In several southern African countries, providing HIV test results to clients of health services and research participants is at the discretion of the client. The fact that there is no requirement for people who test positive to inform others who may benefit from the disclosure is, in my opinion, an important omission in the prevention of HIV in the region. It may be useful to include, at the time of obtaining informed consent, the statement, “Should you test HIV positive, we will encourage you to inform your sexual partners about the test results.”

While it has been demonstrated that MC can be effective, it has yet to be determined why this might be the case. The authors have suggested that perhaps the keratinization that may ensue, more rapid drying of the glans penis after sexual intercourse, and prevention of STIs may be reasons. These are plausible explanations, but it will require separate studies to elucidate the mechanism(s).

The authors suggest that if women were aware of the effectiveness of MC, this would in turn lead them to encourage males to be circumcised. While I agree that all stakeholders ought to be mobilized in promoting an effective HIV intervention, or any public health intervention, the “role” of women, sadly, is minimal in decision making in most parts of the southern African region. But this does not mean that attempts should not be made to involve women.
